# Plasma glial fibrillary acidic protein and neurofilament light chain for the diagnostic and prognostic evaluation of frontotemporal dementia

**DOI:** 10.1186/s40035-021-00275-w

**Published:** 2021-12-10

**Authors:** Nuole Zhu, Miguel Santos-Santos, Ignacio Illán-Gala, Victor Montal, Teresa Estellés, Isabel Barroeta, Miren Altuna, Javier Arranz, Laia Muñoz, Olivia Belbin, Isabel Sala, Maria Belén Sánchez-Saudinós, Andrea Subirana, Laura Videla, Jordi Pegueroles, Rafael Blesa, Jordi Clarimón, Maria Carmona-Iragui, Juan Fortea, Alberto Lleó, Daniel Alcolea

**Affiliations:** 1 Sant Pau Memory Unit, Department of Neurology, Institut d’Investigacions Biomèdiques Sant Pau – Hospital de Sant Pau, Universitat Autònoma de Barcelona, 08041 Barcelona, Spain; 2 Centro de Investigación Biomédica en Red en Enfermedades Neurodegenerativas (CIBERNED), 28031 Madrid, Spain; 3 Autonomous University of Barcelona, 08913 Barcelona, Spain; 4 Fundación Catalana Síndrome de Down, Centre Mèdic Down, 08029 Barcelona, Spain

**Keywords:** Glial fibrillary acidic protein, Neurofilament, Frontotemporal dementia, Plasma biomarkers

## Abstract

**Background:**

Astrocytes play an essential role in neuroinflammation and are involved in the pathogenesis of neurodenegerative diseases. Studies of glial fibrillary acidic protein (GFAP), an astrocytic damage marker, may help advance our understanding of different neurodegenerative diseases. In this study, we investigated the diagnostic performance of plasma GFAP (pGFAP), plasma neurofilament light chain (pNfL) and their combination for frontotemporal dementia (FTD) and Alzheimer’s disease (AD) and their clinical utility in predicting disease progression.

**Methods:**

pGFAP and pNfL concentrations were measured in 72 FTD, 56 AD and 83 cognitively normal (CN) participants using the Single Molecule Array technology. Of the 211 participants, 199 underwent cerebrospinal (CSF) analysis and 122 had magnetic resonance imaging. We compared cross-sectional biomarker levels between groups, studied their diagnostic performance and assessed correlation between CSF biomarkers, cognitive performance and cortical thickness. The prognostic performance was investigated, analyzing cognitive decline  through group comparisons by tertile.

**Results:**

Unlike pNfL, which was increased similarly in both clinical groups, pGFAP was increased in FTD but lower than in AD (all *P* < 0.01). Combination of both plasma markers improved the diagnostic performance to discriminate FTD from AD (area under the curve [AUC]: combination 0.78; pGFAP 0.7; pNfL 0.61, all *P* < 0.05). In FTD, pGFAP correlated with cognition, CSF and plasma NfL, and cortical thickness (all *P* < 0.05). The higher tertile of pGFAP was associated with greater change in MMSE score and poor cognitive outcome during follow-up both in FTD (1.40 points annually, hazard ratio [HR] 3.82, *P* < 0.005) and in AD (1.20 points annually, HR 2.26, *P* < 0.005).

**Conclusions:**

pGFAP and pNfL levels differ in FTD and AD, and their combination is useful for distinguishing between the two diseases. pGFAP could also be used to track disease severity and predict greater cognitive decline during follow-up in patients with FTD.

**Supplementary Information:**

The online version contains supplementary material available at 10.1186/s40035-021-00275-w.

## Introduction

Frontotemporal dementia (FTD) is a progressive neurodegenerative condition characterized by clinical, genetic and neuropathologic heterogeneity. The clinical manifestation of FTD may overlap with psychiatric or other neurodegenerative disorders, such as Alzheimer’s disease (AD). Diagnosis is thus a clinical challenge.

In the past two decades, many efforts have been made to find imaging or fluid biomarkers for FTD [1–6]. In spite of the notable advances, we still do not have pathophysiological markers of FTD to be used in clinical practice. Recently, some studies have shown that the concentrations of neurofilament light chain (NfL), a marker of axonal damage, are increased in the cerebrospinal fluid (CSF) and blood of FTD patients [6]. Moreover, their levels can reflect disease severity and predict clinical progression [1–6]. However, NfL is not specific  for FTD and can be increased in other neurodegenerative diseases, such as AD and Lewy body dementia [6]. There is a considerable overlap of NfL level between different conditions [1–6].

Neuroinflammation and neurodegeneration are highly interrelated processes in neurological diseases [7]. Increased levels of astrogliosis marker glial fibrillary acidic protein (GFAP) in CSF and plasma, have been described in different neurodegenerative diseases [8–19, 20, 23–27]. In AD, recent studies showed that the plasma GFAP (pGFAP) levels are associated with amyloid pathology [15–18]. Furthermore, some studies have suggested that GFAP could be a marker of disease severity [8–22] and be a prognostic marker for progression to AD dementia in cognitively normal older adults [15–22]. Likewise, other studies have reported increased CSF GFAP and pGFAP concentrations in FTD, even though the results are not consistent across studies [10, 12, 13, 23–27]. Although previous studies did not detect changes of blood GFAP level in FTD [10, 12, 13], some studies have observed elevated pGFAP in all different FTD subgroups [24,^25^, ^26^]. Higher pGFAP concentrations have been associated with greater functional impairment and disease severity in two studies [13, 25], but the role of pGFAP in predicting disease progression of FTD and its potential use for diagnosis, either alone or in combination with other biomarkers, remain unclear.

In this study, our primary research aim was to determine the potential use of pGFAP, pNfL or their combination to distinguish patients with FTD from AD and from cognitively normal participants (CN), and to study their association with disease progression in FTD. The secondary aim was to study the correlation of pGFAP with cognition, other biomarkers, and structural measures in neuroimaging.

## Methods

### Study participants and classification

We collected clinical and biomarker information of 211 participants with available plasma samples from the Sant Pau Initiative on Neurodegeneration (SPIN) cohort, a multimodal biomarker platform for the study of neurodegenerative diseases [28]. The participants were classified into the following clinical groups according to the internationally accepted diagnostic criteria: 72 patients with probable FTD-related clinical syndromes: 33 patients with behavioral variant of frontotemporal dementia (bvFTD) [29], 7 semantic variant of primary progressive aphasia (svPPA) [30], 14 nonfluent variant of primary progressive aphasia (nfvPPA) [30] and 18 progressive supranuclear palsy-corticobasal syndrome spectrum (PSP-CBD) [31, 32]. CSF AD biomarkers and/or amyloid PET were taken into account during the diagnostic classification of FTD patients, and those with positive AD biomarkers were excluded from this group. We also included 56 AD patients with evidence of AD pathophysiology [33] either through CSF biomarkers (*n* = 51) or amyloid-PET (*n* = 5), and 83 CN that had normal CSF values of Aβ_42_/Aβ_40_ and pTau181, and neuropsychological evaluation within normal range [28].

Plasma availability was the prerequisite to include the participants in this study. CSF samples were available from 199 participants (94%). A subset of 122 participants (58%) underwent 3 Tesla structural brain MRI. *APOE* genotype was available in 203 participants (96%), and 202 participants (96%) were longitudinally followed up and underwent a comprehensive evaluation. All participants had a Mini-Mental State Examination (MMSE) score and a Global Deterioration Scale of Reisberg score at the time of diagnosis, and repeated measures of MMSE were obtained during the follow-up. In a subset of 45 FTD patients, Frontotemporal Dementia Rating Scale (FTD-FRS) was available at the time of diagnosis.

### Blood and CSF sample analysis

Blood samples were collected in 10-ml EDTA tubes and immediately transferred to our laboratory where they were centrifuged and aliquoted within 2 h after collection. CSF samples were collected on the same day of blood extraction and processed in polypropylene tubes following international recommendations [33]. All samples were processed and aliquoted within the first two hours after lumbar puncture. The plasma and CSF aliquots were stored at − 80 °C until analysis. pGFAP and pNfL concentrations were measured using the SR-X single molecule array. CSF AD core biomarkers (Aβ42, Aβ40, tTau and pTau181) were measured in the fully-automated platform Lumipulse (Fujirebio-Europe), and levels of CSF NfL (Uman Diagnostics) and CSF YKL-40 (MicroVue™, Quidel) were measured by ELISA according to previously reported methods [7, 33]. All samples and calibration curves were measured in duplicate. Intra- and inter-assay coefficients of variations were 5.3% and 9.6%, respectively. The pre-analytical processing protocol for blood and CSF in the SPIN cohort has been described in detail [28, 29].

### Statistical analysis

Chi-square test was used to compare sex and *APOE* ε4 genotype frequency between the groups. Continuous variables are expressed as means and standard deviation. Distributions of demographic and biomarker data were assessed using Shapiro–Wilk’s test and homogeneity of variances was checked by Levene’s test. Biomarker raw values not following a normal distribution were log-transformed to achieve a normal distribution. Age- and sex-adjusted analysis of covariance (ANCOVA) followed by *post-hoc* Tukey’s test was used to compare the pGFAP and pNfL levels between groups. The potential use of pGFAP, pNfL or their combination to distinguish patients with FTD from AD and CN was assessed through areas under the curve (AUC) by receiver operating characteristic (ROC) analyses. ROC curves were compared using DeLong’s test. To study the association of pGFAP with disease progression, we divided participants in 3 tertile groups according to their pGFAP levels. A linear-mixed model was used to assess the association of pGFAP with cognitive decline during the follow-up. We included age, sex, MMSE score at the time of diagnosis, pGFAP tertile and its interaction with time as fixed factors, and modeled random intercepts and slopes at the participant level to account for repeated measures. Multivariate Cox regression analysis adjusting for sex and baseline age and Kaplan Meier curves were performed to analyze the predictive value of pGFAP for significant cognitive impairment (MMSE score < 20). To investigate the relationship of pGFAP level with demographics, cognitive scores, CSF biomarkers and pNfL level, Spearman’s correlations were assessed. Cortical thickness was computed with Freesurfer software (version 5.1) [35] (https://surfer.nmr.mgh.harvard.edu/fswiki), and its correlation with pGFAP was assessed using the general linear model, including age and sex as fixed factors.

Statistical significance for all tests was set at 5% (*P* = 0.05) and corrected for multiple comparisons. All statistical analyses were performed using packages “psych” (v. 2.0.8), “ggplot2” (v. 3.3.3), “pROC” (v. 1.16.2), “lmerTest” (v. 3.1-3), “nlme” (v. 3.1-148), “multcomp” (v. 1.4-13), “survival” (v. 3.2-11) and “survminer” (v. 0.4.9) as implemented in R statistical software version R 4.0.2 (http://www.R-project.org).

## Results

### Demographics and clinical data

Table 1 shows the demographics and clinical data, CSF and plasma biomarker levels in the FTD, AD and CN groups. Controls were significantly younger than symptomatic patients, but there was no difference in age between the symptomatic groups. Controls had more years of education than the symptomatic groups, and there were no differences in sex between the groups. *APOE*ε4 genotype was more frequent in the AD group. Both baseline and last MMSE scores were lower in the disease groups compared with the control group. Within the FTD subgroups, the baseline and last MMSE scores were lower in svPPA compared to other subgroups. The mean follow-up period was 3.4 (± 2.3) years in FTD, 3.9 (± 2) in AD and 4 (± 1.7) in CN. The follow-up period did not differ between the groups.

**Table 1:** Demographics and clinical characteristics, CSF and plasma biomarker concentrations in CN, FTD and AD groups

	CN	FTD					AD
		bvFTD	nfvPPA	svPPA	PSP-CBD	All FTD	
*n*	83	33	14	7	18	72	56
Age, years	58 (8.5)^b,c^	68.3 (10.6)^a^	72.8 (5.6)^a^	73.1 (11.1)^a^	72.9 (5.4)^a^	70.8 (8.9)^a^	70.8 (7.2)^a^
Sex							
Female	49	10	9	4	11	34	31
Male	34	23	5	3	7	38	25
*APOE* ε4 status							
ε4+	23^c^	3^c^	3^c^	2^c^	2^c^	10^c^	27^a^^,b,d^^−^^g^
ε4−	57^c^	29^c^	11^c^	5^c^	14^c^	59^c^	27^a^^,b,d^^−^^g^
Follow up time, years	3.9 (1.7)	4.1 (2.5)	3.1 (2.3)	4.8 (1.7)	1.9 (1)	3.4 (2.3)	4 (2)
Education, years	15.4 (4.1)^b−g^	12.9 (5.6)^a^	13.3 (4.9)^a^	14.8 (5.2)^a^	11.3 (3.7)^a^	12.7 (5)^a^	10.4 (4.7)^a^
Baseline MMSE score	29.2 (1)^b−g^	25.1 (4.2)^a,f^	24.1 (6.8)^a,f^	16.5 (12.2)^a,c^^−^^e,g^	22.6 (6.3)^a,f^	23.5 (6.5)^a^	23.7 (3.8)^a^
Last MMSE score	29.3 (1)^b−g^	16.6 (10)^a,f^	14 (12.2)^a,f^	0.4 (0.9)^a,c^^−^^e,g^	11.6 (12.4)^a^	13.3 (11.4)^a^	15.5 (9.5)^a^
pGFAP, pg/ml	134.3 (45.4)^b−d,f−g^	193.1 (100.1)^a^	224.6 (77.2)	159.3 (279.1)^a^	271.2 (148.2)^a^	234.9 (141.9)^a,b^	319.8 (135.1)^a,c^
pNfL, pg/ml	18 (20.2)^b^^−^^e,g^	41.9 (60.1)^a^	34.9 (17.2)^a^	31.3 (14.5)	34.3 (18.1)^a^	37.6 (42.3)^a^	26.5 (13.3)^a^
CSF NfL, pg/ml	494.8 (274.3)^b,c,f,g^	1436.3 (930.6)^a^	1944.5 (1146.7)	2394.6 (637.8)^a^	1739.3 (1607.3)^a^	1654.1 (1187.6)^a,c^	1286.4 (1136.9)^a,b,f^
CSF YKL-40, ng/ml	190.3 (49.4)^b,c^	265.8 (64.7)^a^	274.84 (61)^a^	311.8 (37.1)^a^	262.3 (79.3)	268.2 (63.7)^a^	287.3 (68.1)^a^
CSF Aβ42/Aβ40	0.099 (0.016)^c^	0.098 (0.015)^c^	0.092 (0.012)^c^	0.103 (0.0002)^c^	0.095 (0.012)^c^	0.097 (0.013)^c^	0.046 (0.0099)^a,b,d^^−^^g^
CSF tTau, pg/ml	264 (103.9)^c^	388.9 (182.3)^c^	275.4 (144.5)^c^	367.5 (0.7)	276.1 (122.7)^c^	332.7 (162)^c^	774.2 (390.8)^a,b,d,f,g^
CSF pTau, pg/ml	39.8 (17.8)^c^	45.7 (16.1)^c^	42.8 (21.9)^c^	47.2 (10)	33.9 (15)^c^	42.1 (16.4)^c^	121.2 (71)^a,b,d,f,g^

MMSE, Mini-Mental State Examination; pGFAP, plasma glial fibrillary acidic protein; pNfL, plasma neurofilament light chain; CN, cognitivelly normal participants; FTD, Frontotemporal dementia; AD, Alzheimer disease; bvFTD, behavioral variant of frontotemporal dementia; nfvPPA, nonfluent variant of primary progressive aphasia; svPPA, semantic variante of primary progressive aphasia; PSP-CBD, progressive supranuclear palsy-corticobasal syndrome spectrum

^a^Different from Control (*P* < 0.05)

^b^Different from FTD (*P* < 0.05)

^c^Different from AD (*P* < 0.05)

^d^Different from bvFTD (*P* < 0.05)

^e^Different from fnvPPA (*P* < 0.05)

^f^Different from svPPA (*P* < 0.05)

^g^Different from PSP-CBD (*P* < 0.05). Data are shown as mean (standard deviation)

Age correlated with the pGFAP level (Rho = 0.35, *P* < 0.001) in the control group, but not with the pNfL level. No difference in pGFAP level was found between male and female participants. pNfL levels were higher in males in the whole sample (*P* = 0.05) and in the AD group (*P* = 0.04), but not in the CN or the FTD group.

### Plasma GFAP and NfL concentrations differ between FTD and AD

**Fig. 1:**
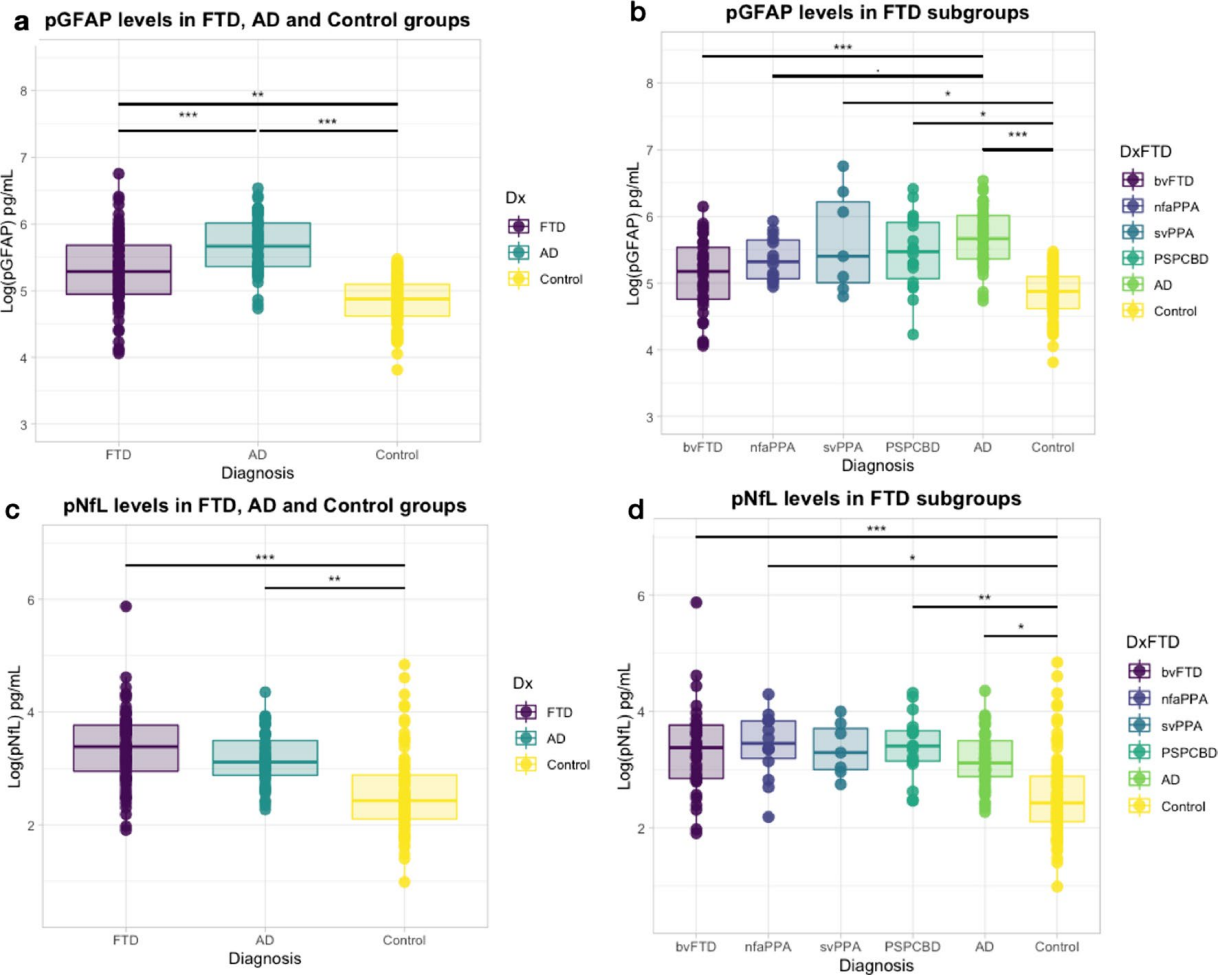
Comparison of pGFAP and pNfL in different groups. **a** pGFAP in the FTD, AD and cognitively normal groups; **b** pGFAP concentration in all FTD subgroups, AD and cognitively normal groups; **c** pNfL concentration in the FTD, AD and cognitively normal groups; **d** pNfL concentration in all FTD subgroups, AD and cognitively normal groups. *P < 0.05, **P < 0.01, ***P < 0.001. pGFAP, plasma glial fibrillary acidic protein; pNfL, plasma neurofilament light chain; CN, cognitively normal participants; FTD, frontotemporal dementia; AD, Alzheimer’s disease

After adjusting for age and sex, pGFAP and pNfL levels differed between FTD and AD patients (Fig. 1). The level of pGFAP was higher in patients with FTD (234.9 ± 141.9 pg/ml) than in CN (134.3 ± 45.4 pg/ml, *P* = 0.0008) but lower than in AD (319.8 ± 135.1 pg/ml, *P* < 0.001). Within the FTD subgroups, the pGFAP level was significantly higher in svPPA and PSP-CBD compared with controls (*P* = 0.03), and significantly lower in bvFTD than in AD (*P* < 0.001) (Fig. 1a, b).

As expected, the pNfL levels were higher in both FTD (37.6 ± 42.3 pg/ml, *P* < 0.001) and AD (26.5 ± 13.3 pg/ml, *P* = 0.004) groups compared to CN (18 ± 20.2 pg/ml). The pNfL levels were increased in all FTD subgroups (*P* < 0.05) compared to CN, except in svPPA, which showed a similar trend as other FTD subgroups but did not reach statistical significance (Fig. 1c, d).

### Diagnostic accuracy of plasma biomarkers and their combination to differentiate between FTD, AD and CN

**Fig. 2:**
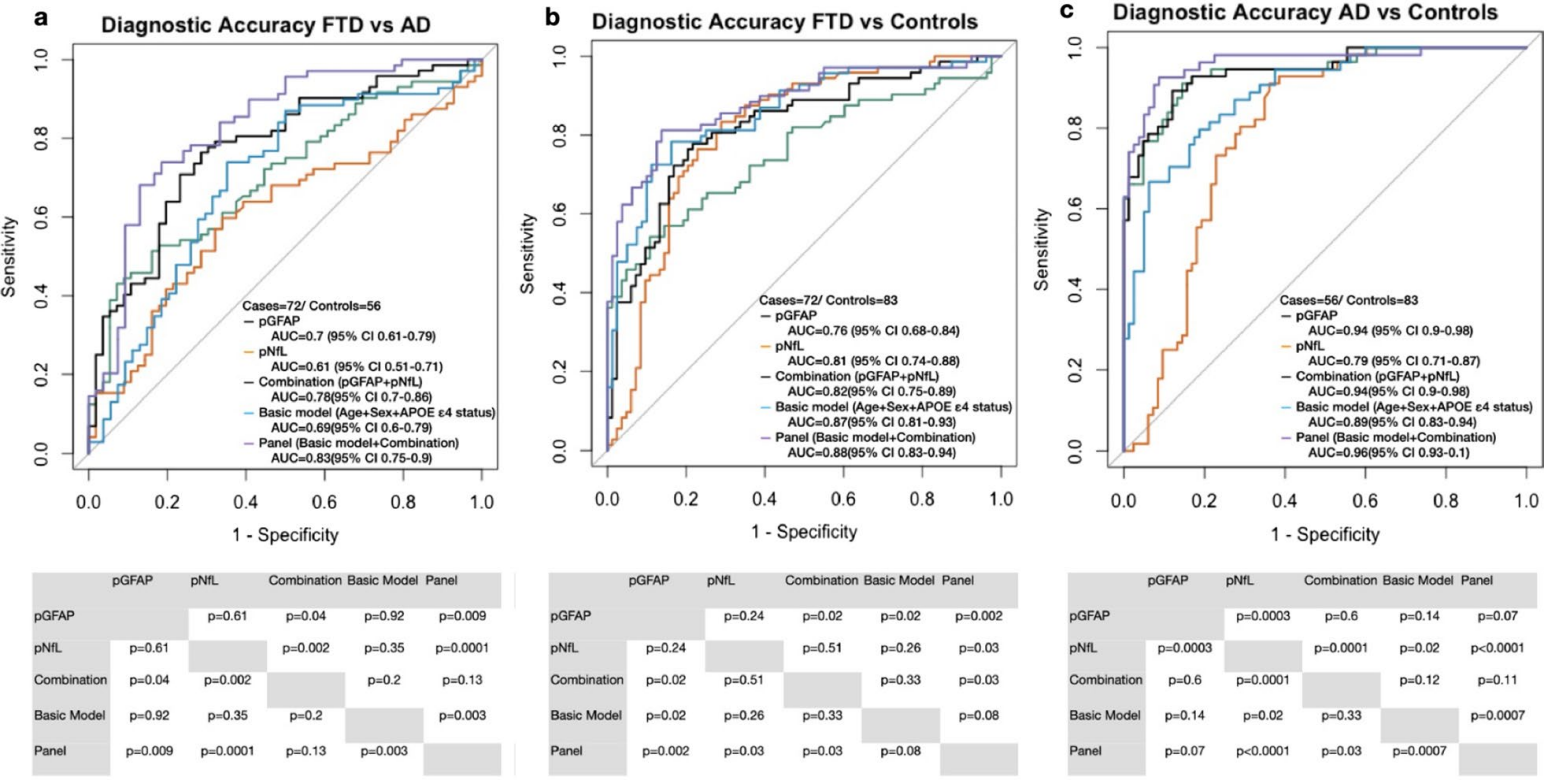
Receiver operating characteristic curves (ROC) of pGFAP, plasma neurofilament light chain, their combination, a basic model with risk factors (age, sex and APOE), and a combination of plasma biomarkers with additional risk factors to discriminate FTD from AD (**a**), FTD from normal cognitively normal (**b**), and AD from cognitively normal (**c**). AUC, area under the curve; pGFAP, plasma glial fibrillary acidic protein; pNfL, plasma neurofilament light chain; CN, cognitively normal participants; FTD, frontotemporal dementia; AD, Alzheimer’s disease

We next explored the diagnostic performance of pGFAP, pNfL and their combination using logistic regression to discriminate across the different groups. The basic model included age, sex and *APOEε4* allele status. The diagnostic performance of this basic model was then compared with a panel including the variables in the basic model together with pGFAP and pNfL. ROC curve analysis (Fig. 2) showed that the combination of pGFAP and pNfL had higher accuracy than the two plasma markers separately in differentiating FTD from AD (combination: AUC 0.78 [95%CI 0.70–0.86]; pGFAP: AUC 0.7 [95%CI 0.61–0.71]; pNfL: AUC 0.61 [95%CI 0.51–0.71], *P* = 0.04 and *P* = 0.002, respectively) and for the discrimination between FTD and CN (combination: AUC 0.82 [95%CI 0.75–0.89]; pGFAP: AUC 0.76 [95%CI 0.68–0.84]; pNfL: AUC 0.81 [95%CI 0.74–0.88], *P* = 0.02 and *P* = 0.51, respectively). None of the two plasma markers, individually or in combination, showed higher accuracy than the basic model. However, the addition of pGFAP and pNfL to the basic model (panel model) led to better diagnostic performance than the basic model both in differentiating FTD from AD (AUC 0.83 [95%CI 0.75–0.9] *vs* AUC 0.69 [95%CI 0.6–0.79], *P* = 0.003) and AD from CN (AUC 0.96 [95%CI 0.93–1] *vs* AUC 0.89 [95%CI 0.83–0.95], *P* = 0.0007). For differentiation of FTD from CN, the panel showed highest AUC compared to other methods (all *P* < 0.05), but not compared to the basic model (AUC 0.88 [95%CI 0.83–0.94] *vs* AUC 0.87 [95%CI 0.81–0.93], *P* = 0.08).

Taking into account that pGFAP increases with age, we repeated the same analyses in an age-matched subgroup. In this new comparison, similar results were observed except in the comparison between FTD and CN, in which both pNfL and the combination showed higher diagnostic performance compared to this basic model (Combination: AUC 0.8 [95%CI 0.7–0.9]; pNfL: AUC 0.8 [95%CI 0.7–0.9]; Basic model: AUC 0.59 [95%CI 0.47–0.72], *P* = 0.05 and *P* = 0.005) (Additional file 1).

### pGFAP correlated with other fluid biomarkers, cognitive and functional scores and neuroimaging

**Fig. 3:**
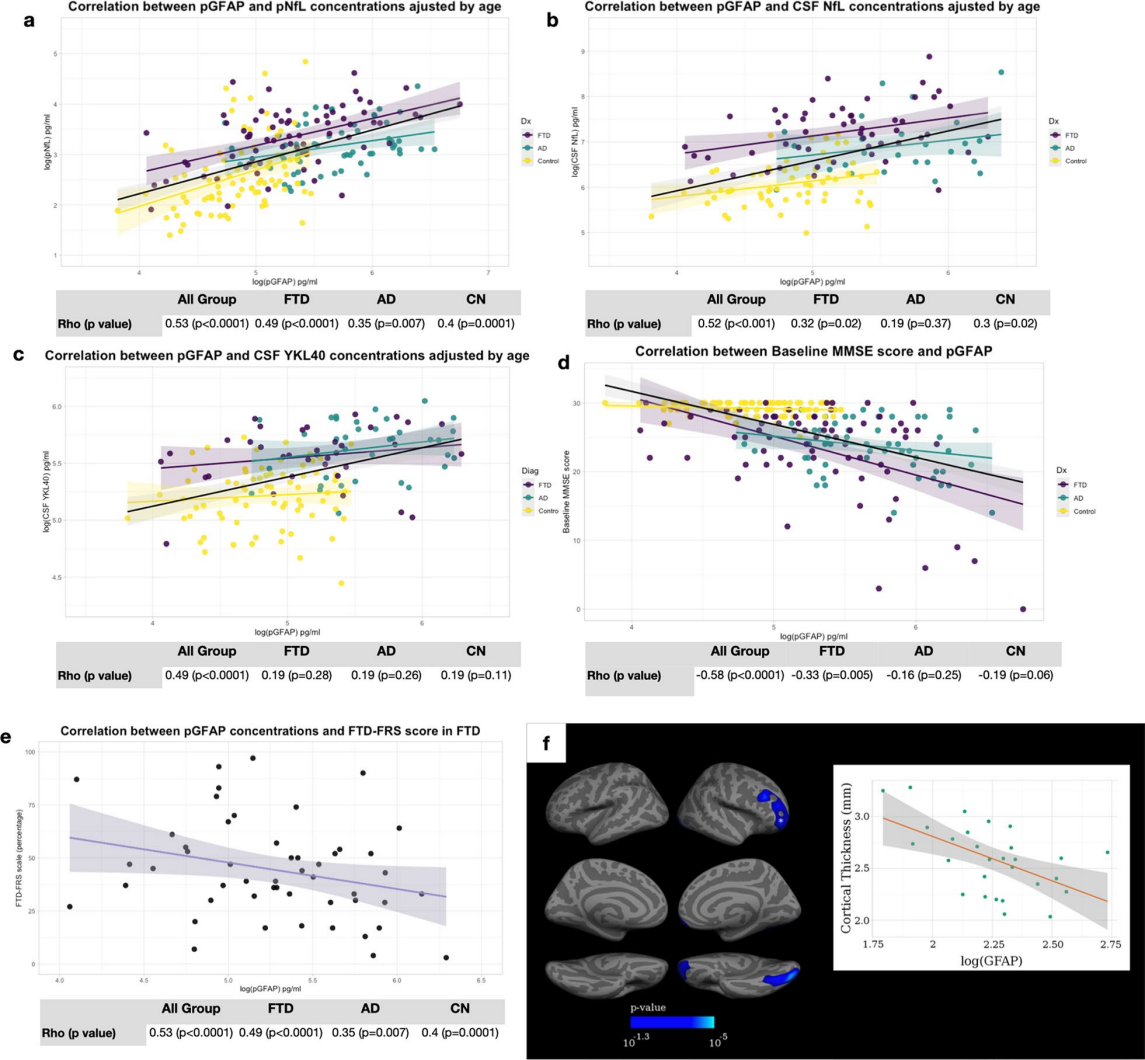
Correlation of pGFAP with plasma and CSF neurofilament light chain (**a**, **b**), CSF YLK-40 (**c**), and baseline MMSE score (**d**) in FTD, AD and cognitively normal participants. Correlation of pGFAP with baseline FTD-FRS score (**e**) and cortical thickness in FTD (**f**, *n* = 29). Blue regions in **f** represent a direct correlation. For illustrative purposes, a scatterplot shows the individual log(pGFAP) and the value of cortical thickness in the corresponding cortical region (marked with an asterisk) (**f**). *P*-value 10^−1.3^ = 0.05. All correlation analyses were adjusted by age and sex. pGFAP, plasma glial fibrillary acidic protein; pNfL, plasma neurofilament light chain; CN, cognitively normal participants; FTD, frontotemporal dementia; AD, Alzheimer’s disease

In the whole sample, pGFAP level correlated with pNfL (Rho = 0.53, *P* < 0.001), CSF NfL (Rho = 0.52, *P* < 0.001) and CSF YKL-40 levels (Rho = 0.49, *P* < 0.001) after adjusting for age and sex. To avoid group effects on the correlation assessment, we studied correlations of these biomarkers within groups. The pGFAP level correlated with plasma and CSF NfL in FTD (Rho = 0.49, *P* < 0.001; Rho = 0.32, *P* < 0.02) and in CN (Rho = 0.4, *P* < 0.001; Rho = 0.3, *P* = 0.016), but not with CSF YKL-40. By contrast, in the AD group, pGFAP level correlated with pNfL level only (Rho = 0.35, *P* = 0.007) (Fig. 3a–c).

The pGFAP level was significantly correlated with AD-specific CSF biomarkers, such as the Aβ42/Aβ40 ratio, amyloid-PET centiloid, total tau and phosphorylated tau in the whole sample, but these correlations were not significant within diagnostic subgroups (Additional file 2).

After adjusting for age and sex, pGFAP was also significantly associated with the baseline MMSE scores in the whole sample (Rho =  − 0.58, *P* < 0.001) and in the FTD group (Rho =  − 0.33, *P* = 0.005), but not in the AD or the CN group (Fig. 3d). In the FTD group, after adjusting for age, higher pGFAP concentrations were associated with lower FTD-FRS scores (*r* =  − 0.28, *P* = 0.046, Fig. 3e).

We assessed the correlation between pGFAP and cortical thickness in a subset of 122 participants (29 FTD, 25 AD and 68 CN) with structural MRI suitable for quantitative analyses. After adjusting for age, sex and neuroimaging acquisition center, vertex-wise regression analysis showed a significant association between pGFAP and cortical thickness in the orbitofrontal and occipital pole regions (Fig. 3f, P < 0.05) in the FTD group. In the AD and the CN groups, no correlation between pGFAP concentration and cortical thickness was found (not shown).

### Baseline pGFAP level predicts cognitive decline

To investigate the prognostic performance of pGFAP levels, we divided pGFAP levels in tertiles within each symptomatic group: low (< 154 pg/ml in FTD and < 228 pg/ml in AD), medium (154–240 pg/ml in FTD and 228–366 pg/ml in AD) and high (> 240 pg/ml in FTD and > 366 pg/ml in AD).

**Fig. 4:**
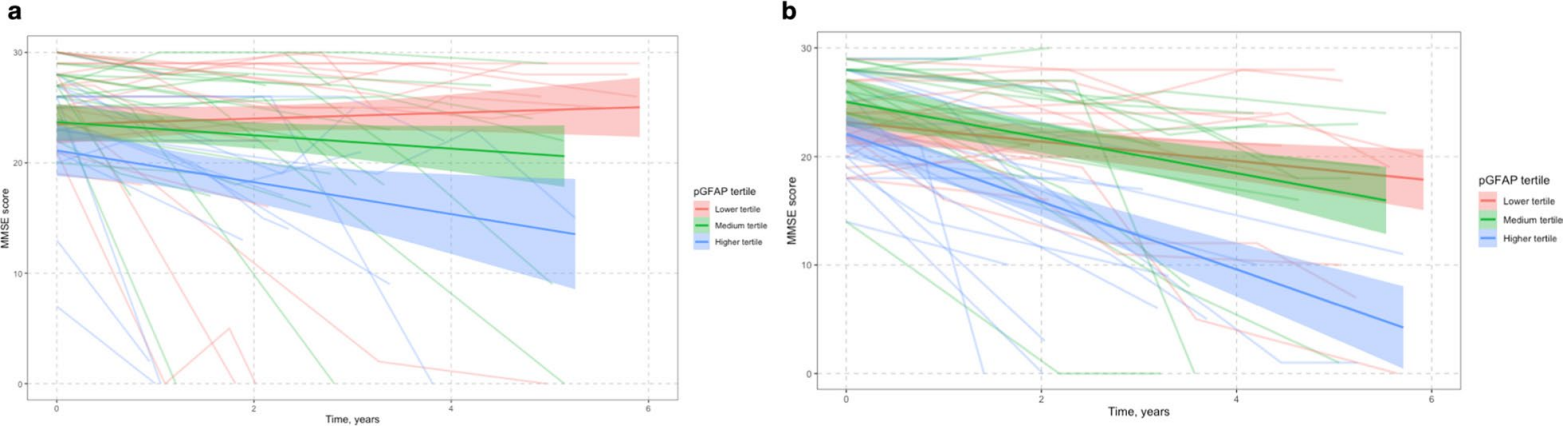
Relationship between the baseline tertile of pGFAP and longitudinal changes of cognitive score (MMSE score) in FTD (**a**) and AD (**b**). Cognitive changes were estimated through linear mixed effects regression models adjusted for age and baseline MMSE score. Red lines show the lowest tertile (below 154 pg/ml in FTD and below 228 pg/ml in AD), green lines show the medium tertile (154–240 pg/ml in FTD and 226–366 pg/ml in AD), and blue lines show the highest tertile of pGFAP level (above 240 pg/ml in FTD and above 366 pg/ml in AD). Shaded areas indicate the 95% confidence interval for predicted cognitive scores. pGFAP, plasma glial fibrillary acidic protein; pNfL, plasma neurofilament light; CN, cognitivelly normal participants; FTD, frontotemporal dementia; AD, Alzheimer’s disease

Linear-mixed model analysis was used to assess the relationship between the baseline pGFAP level and cognitive decline measured by changes in MMSE score during follow-up. After adjusting for age, sex and baseline MMSE score, patients in the highest pGFAP tertile showed a greater change in MMSE score compared to those in the lowest tertile in both FTD (mean loss of 1.40 points annually, *P* = 0.003) and AD (mean loss of 1.20 points annually, *P* < 0.001) (Fig. 4a, b).

**Fig. 5:**
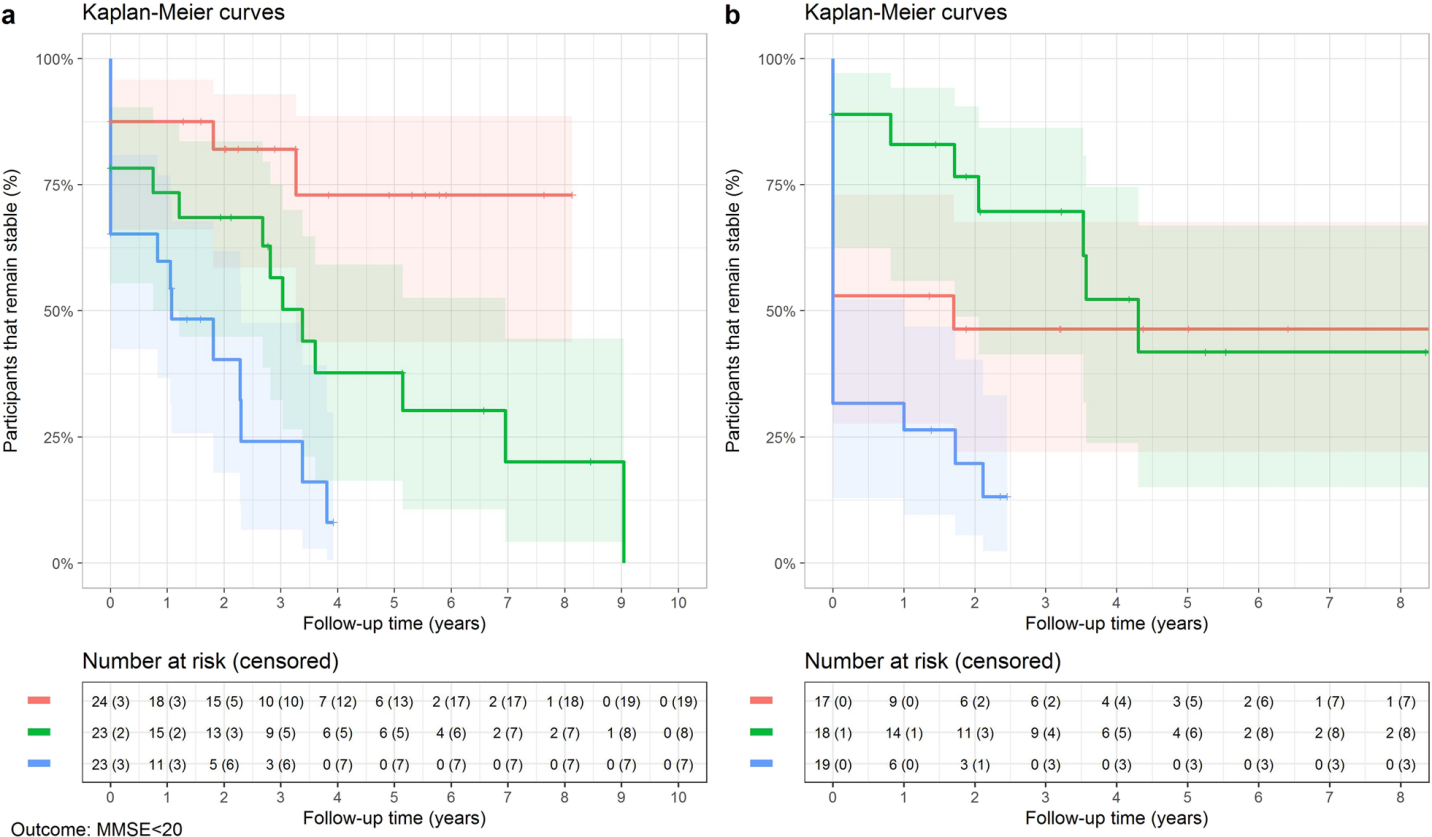
Kaplan Meier curves of clinical progression to significant cognitive impairment (MMSE score < 20) in FTD (**a**) and AD (**b**) individuals with low, medium or high baseline pGFAP level. Red lines show the lowest tertile (below 154 pg/ml in FTD and below 228 pg/ml in AD), green lines show medium tertile (154–240 pg/ml in FTD and 226–366 pg/ml in AD), and blue lines show the highest tertile of biomarker concentrations (above 240 pg/ml in FTD and above 366 pg/ml in AD). In FTD, the median time of progression to significant cognitive impairment was 3.4 (0.8**–**7.0) years in the medium-tertile pGFAP group and 1.1 (0–2.3) years in the high-tertile pGFAP group (Log-rank test, *P* < 0.0001). pGFAP, plasma glial fibrillary acidic protein; FTD, frontotemporal dementia; AD, Alzheimer’s disease

Kaplan Meier curves (Fig. 5) and Cox regression analyses including baseline age and sex as covariates were used to assess progression to moderate cognitive impairment (MMSE score < 20). Compared to the lowest tertile, the highest tertile of pGFAP was associated with increased risk of poor cognitive outcome both in FTD and in AD (1.40 points annually, HR = 3.82 in FTD; 1.20 points annually, HR = 2.26 in AD, both *P* < 0.001).

## Discussion

In this study, we found that pGFAP was increased in FTD, and that its level differs from that in AD patients. pGFAP also correlated with neuronal damage biomarkers, cognitive and functional scores and structural imaging measures in FTD. Importantly, a higher pGFAP level was associated with a greater change in MMSE score during follow-up and predicted progression to moderate cognitive impairment in FTD. Our findings suggest that pGFAP could be a useful marker of disease severity and for prognostic assessment in FTD, in addition to its role in AD.

Emerging evidence suggests that astrocytes play an essential role in neuroinflammation and are involved in the pathophysiology of several neurological diseases [34, 36, 38]. Biomarkers that track astrocyte biology, such as GFAP, have been investigated over the past few years in neurodegenerative diseases. In AD, previous studies have shown that reactive astrocytes are closely associated with senile plaques and neurofibrillary tangles [37–39]. Elevated concentrations of GFAP in CSF and blood have been observed in AD, and it has been reported that its levels correlate with disease severity [8–16, 19]. Recent studies have shown that the elevated CSF and plasma GFAP are associated with amyloid pathology, also in cognitively unimpaired subjects [15–20]. In addition, astrogliosis is also recognized in FTD. Astrogliosis is mainly confined to concrete regions such as the frontal cortex and the hippocampus [40, 41]. Interestingly, increased CSF and plasma GFAP levels have been reported in both sporadic and genetic FTD cohorts [10–12, 14, 23]. These findings suggest that pGFAP may be a valuable tool in FTD diagnosis. According to this hypothesis, various studies have observed elevated pGFAP concentration in different FTD subgroups and both in presenile and late-onset bvFTD cases [24, 25, 27]. In addition, one study in genetic FTDs found increased pGFAP concentration in progranulin-associated symptomatic FTD patients [23]. Here we report an increased pGFAP level in FTD patients, which was, however, lower than that of AD. Our results are in line with previous reports of higher concentrations of pGFAP in AD compared to non-AD dementias [13, 18]. However, other studies found no differences between groups [20, 24]. These discrepancies could be explained by the heterogeneity of the patient composition: some studies included different types of dementias in the non-AD dementia group and little was known about the function of pGFAP in such contexts, such as vascular dementia or dementia with Lewy bodies. Our results support previous clinical-pathological findings that in FTD, astrocytic activation is at a lower extent and is more confined to specific brain regions than that in AD [40, 41]. On the other hand, the increase in pNfL, a marker of axonal damage, suggests a similar degree of neurodegeneration in FTD compared to AD. One possible explanation for the differences in pGFAP levels but not in pNfL between FTD and AD is that FTD and AD are characterized by different neuroinflammatory patterns, with different degrees of astroglial activation in different subpopulations but leading to a similar degree of neuronal degeneration. Similar to a previous study [24], we found that the pGFAP level tended to be different across the subtypes of the FTD spectrum, being significantly higher in svFTD and PSP-CBD compared to CN. This finding could indicate differences in the degree of astrocytic degeneration in different clinical phenotypes [23].

The current study showed that pGFAP had an acceptable performance to discriminate FTD from AD (AUC = 0.7) and CN (AUC = 0.76). These accuracies were somewhat lower than the values reported in two recent studies using pGFAP to identify bvFTD from AD (AUC = 0.85) and frontotemporal lobar degeneration (FTLD) from primary psychiatric disorders (AUC = 0.82) [13, 27]. These discrepancies could be attributed to differences in cohort characteristics and heterogeneity due to different composition of FTD subtypes. An interesting finding was that, contrary to pNfL, which tend to be higher in FTD compared to AD, the pGFAP level was significantly lower in FTD, suggesting the potential diagnostic utility of their combination. Indeed, the combination of pGFAP with pNfL improved the diagnostic performance in distinguishing FTD from AD and CN. Moreover, the incorporation of age, sex and *APOEε4* allele status improved the diagnostic performance to an AUC of 0.83 in differentiating FTD from AD and an AUC of 0.89 in differentiating FTD from CN. In addition, pGFAP was particularly promising in identifying AD, consistent with previous observations [13, 15, 17–20]. Taken together, this study suggests that the combination of plasma astrocytic and neuronal markers could be relevant in FTD.

We observed that the pGFAP level increased with age in controls, similar to what has been observed in previous studies [13, 17, 23, 25], and we took age into account when interpreting this marker. Consistent with various recent reports [15, 17, 21, 23], pGFAP significantly correlated with CSF and plasma NfL, suggesting that glial activation and neuroaxonal degeneration are correlated. Previous studies have reported a weak correlation between CSF GFAP and YKL-40 levels in neurodegenerative diseases [11, 12]. Similarly, we did not find significant correlation between pGFAP and CSF YKL-40 levels. This finding supports the hypothesis that different astrocyte subpopulations or different spatial distributions are involved in the pathophysiology of FTD and AD [12, 13, 37, 38–41].

The association between elevated pGFAP level, disease severity and prognostic markers in AD has been reported before [15, 17–22]. Previous studies reported that the serum GFAP has potentials to predict future conversion to dementia in cognitively normal individuals, not only to AD, but also to other dementias including FTD [16, 17, 22]. However, pGFAP as a marker of disease severity and prognosis in FTD has seldom been investigated [13, 25, 26]. In the current study, after adjusting for age and sex, pGFAP remained significantly correlated with cognitive and imaging measures in FTD. Higher levels of pGFAP have been shown to be associated with lower brain volumes in *GRN* and *C9orf72* presymptomatic carriers [23], and their association with smaller hippocampal volume in svPPA [26] and temporal atrophy in FTLD has been reported [27]. To our knowledge this is the first study to show that higher concentrations of pGFAP are also associated with orbitofrontal cortical thickness in the whole FTD group. In line with other reports in AD patients [16, 20, 22], we observed that pGFAP predicted faster cognitive decline both in FTD and AD. This is the first report of the association between pGFAP and cognitive decline in FTD; we found a higher rate of cognitive decline among those with pGFAP level > 240 pg/ml. Surprisingly, the relationship between pGFAP and disease severity in AD was not confirmed in our cohort. One of the considerations when comparing our study with the previous ones is that other studies included both Aβ+ and Aβ− subjects [15, 22], whereas in our study the correlation analysis was performed in disease subgroups. Previous studies have found that pGFAP is increased in early stages of AD [15–22] and does not differ between disease stages [19, 20], which could explain the lack of association with imaging measures in the AD subgroup. Consistent with previous studies [2, 7, 17], our results show that the glial biomarker increases also in FTD, in addition to AD, and its level increases later in the disease course in FTD than in AD, when the disease is more advanced.

The strength of our study is the relatively large sample size, the inclusion of several FTD subgroups, regular follow-up of the participants and the use of multimodal approach, with inclusion of clinical measures, plasma and CSF assessments, and structural imaging. Additionally, all blood-based biomarkers were collected using the same standard operating procedures and measured following a harmonized protocol. This study also has some limitations, in that it did not include serial longitudinal measures of pGFAP, lacked neuropathological confirmation, and excluded AD copathologies in FTD groups.

## Conclusion

In conclusion, our study shows that the pGFAP concentration may be useful in FTD and could improve its diagnosis. Furthermore, our data support that pGFAP is not an exclusive marker in AD, but also plays an essential role in other amyloid-independent processes involved in FTD. pGFAP could be used as a marker for FTD severity and prognosis.

## Supplementary Information

**Additional file 1**. Diagnostic accuracy of plasma biomarkers and their combination to differentiate between FTD, AD and CN in age-matched group

**Additional file 2**. The correlation of pGFAP with CSF AD-specific biomarkers

## Data Availability

The datasets used and/or analyses during the current study are available from the corresponding author on reasonable request.

## Abbreviations

AD: Alzheimer’s disease
AUC: Area under the curve
bvFTD: Behavioral variant of frontotemporal dementia
CN: Cognitively normal participants
CSF: Cerebrospinal fluid
FTD: Frontotemporal dementia
FTLD: Frontotemporal lobar degeneration
FTD-FRS: Frontotemporal Dementia Rating Scale
GFAP: Glial fibrillary acidic protein
HR: Hazard ratio
MMSE: Mini-Mental State Examination
NfL: Neurofilament light chain
nfvPPA: Nonfluent variant of primary progressive aphasia
PET: Positron emission tomography
pGFAP: Plasma Glial fibrillary acidic protein
pNfL: Plasma neurofilament-light
PSP-CBD: Progressive supranuclear palsy-corticobasal syndrome spectrum
ROC: Receiver operating characteristic
SPIN: Sant Pau Initiative on Neurodegeneration
svPPA: Semantic variant of primary progressive aphasia
