# Correction: Plasma glial fibrillary acidic protein and neurofilament light chain for the diagnostic and prognostic evaluation of frontotemporal dementia

**DOI:** 10.1186/s40035-023-00351-3

**Published:** 2023-05-09

**Authors:** Nuole Zhu, Miguel Santos-Santos, Ignacio Illán-Gala, Victor Montal, Teresa Estellés, Isabel Barroeta, Miren Altuna, Javier Arranz, Laia Muñoz, Olivia Belbin, Isabel Sala, Maria Belén Sánchez-Saudinós, Andrea Subirana, Laura Videla, Jordi Pegueroles, Rafael Blesa, Jordi Clarimón, Maria Carmona-Iragui, Juan Fortea, Alberto Lleó, Daniel Alcolea

**Affiliations:** 1 Sant Pau Memory Unit, Department of Neurology, Institut d’Investigacions Biomèdiques Sant Pau – Hospital de Sant Pau, Universitat Autònoma de Barcelona, 08041 Barcelona, Spain; 2 Centro de Investigación Biomédica en Red en Enfermedades Neurodegenerativas (CIBERNED), 28031 Madrid, Spain; 3 Autonomous University of Barcelona, 08913 Barcelona, Spain; 4 Fundación Catalana Síndrome de Down, Centre Mèdic Down, 08029 Barcelona, Spain

**Correction: Translational Neurodegeneration (2021) 10:50** 10.1186/s40035-021-00275-w

Following publication of this article [1], the below funding information is missing.

European Union's Horizon 2020, 'MES-CoBraD' (H2020-SC1-BHC-2018-2020/GA 965422 to JF).

The original article [1] has been corrected.
