# Supplementary material for: Plasma glial fibrillary acidic protein and neurofilament light chain for the diagnostic and prognostic evaluation of frontotemporal dementia
Source: Transl Neurodegener. 2021 Dec 10;10:50. doi: 10.1186/s40035-021-00275-w (PMC8662866; doi:10.1186/s40035-021-00275-w)
Supplement: Supplementary file 1 — Additional file 1. Diagnostic accuracy of plasma biomarkers and their combination to differentiate between FTD, AD and CN in age-matched group [file 40035_2021_275_MOESM1_ESM.pdf]

Additional data 1: Diagnostic accuracy of plasma biomarkers and their combination to differentiate between FTD, AD and CN in age-matched group

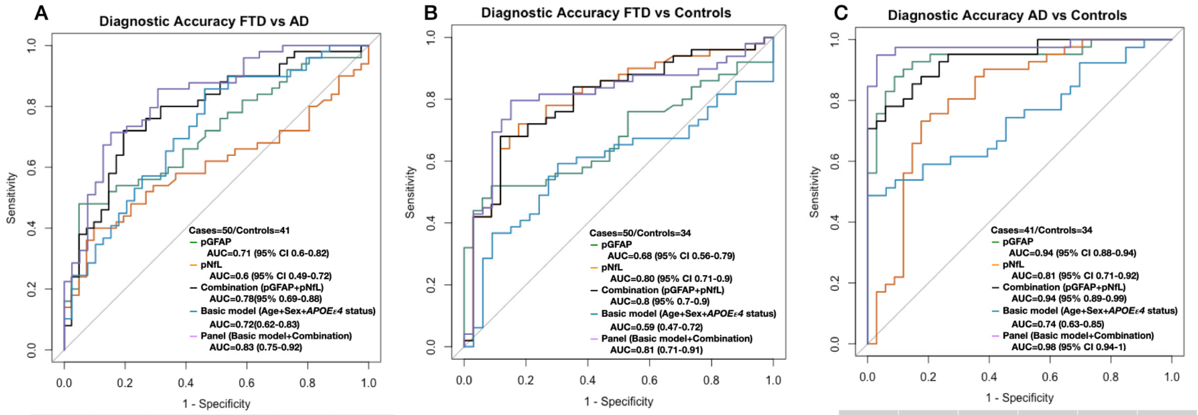

	pGFAP	pNfL	Combination	Basic model	Panel
pGFAP		p=0.24	p=0.06	p=0.7	p=0.013
pNfL	p=0.24		p=0.006	p=0.23	p=0.001
Combination	p=0.06	p=0.006		p=0.47	p=0.18
Basic model	p=0.7	p=0.23	p=0.47		p=0.02
Panel	p=0.013	p=0.001	p=0.18	p=0.02	

	pGFAP	pNfL	Combinatio	Basic model	Panel
pGFAP		p=0.04	p=0.03	p=0.3	p=0.004
pNfL	p=0.04		p=0.53	p=0.005	p=0.92
Combinatio	p=0.03	p=0.53		p=0.05	p=0.75
Basic model	p=0.3	p=0.005	p=0.05		p=0.006
Panel	p=0.004	p=0.92	p=0.75	p=0.006	

	pGFAP	pNfL	Combination	Basic model	Panel
pGFAP		p=0.037	p=0.88	p=0.004	p=0.06
pNfL	p=0.037		0.01	p=0.45	p=0.003
Combination	p=0.88	p=0.01		p=0.003	p=0.04
Basic model	p=0.004	p=0.45	p=0.003		p<0.0001
Panel	p=0.06	p=0.003	p=0.04	p<0.0001	

Receiver operating characteristic curves (ROC) for plasma GFAP, plasma neurofilament light, their combination, a basic model with risk factors (age, sex and APOE) and a combination of plasma biomarkers with additional risk factors to assess the accuracy to discriminate FTD from AD (A), FTD from normal cognitively normal (B), and AD from cognitively normal (C) in age matched group. Abbreviations: AUC = area under the curve; pGFAP = plasma Glial fibrillary acidic protein; pNfL = plasma Neurofilament light; FTD = Frontotemporal dementia; AD = Alzheimer’s Disease; CN = cognitively normal participants
