# Supplementary material for: Plasma glial fibrillary acidic protein and neurofilament light chain for the diagnostic and prognostic evaluation of frontotemporal dementia
Source: Transl Neurodegener. 2021 Dec 10;10:50. doi: 10.1186/s40035-021-00275-w (PMC8662866; doi:10.1186/s40035-021-00275-w)
Supplement: Supplementary file 2 — Additional file 2. The correlation of pGFAP with CSF AD-specific biomarkers [file 40035_2021_275_MOESM2_ESM.pdf]

Additional data 2: The correlation of pGFAP with CSF AD-specific biomarkers

A

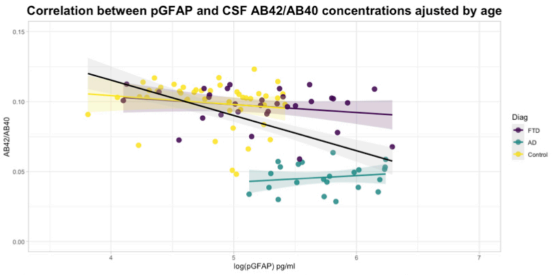

	All Group	FTD	AD	CN
Rho (p value)	-0.56 (p<0.0001)	-0.18 (p=0.32)	0.18 (p=0.4)	-0.26 (p=0.07)

B

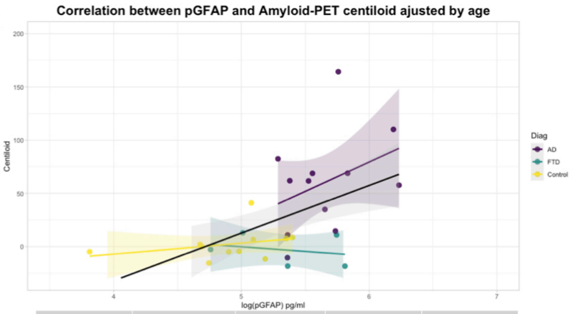

	All Group	FTD	AD	CN
Rho (p value)	0.56 (p=0.002)	0.14 (p=0.79)	0.29 (p=0.37)	0.53 (p=0.096)

C

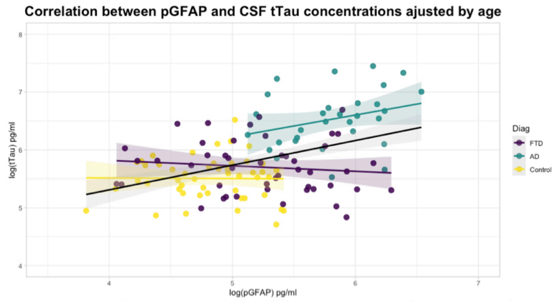

	All Group	FTD	AD	CN
Rho (p value)	0.38 (p<0.001)	-0.15 (p=0.3)	0.29 (p=0.12)	-0.001 (p=0.997)

D

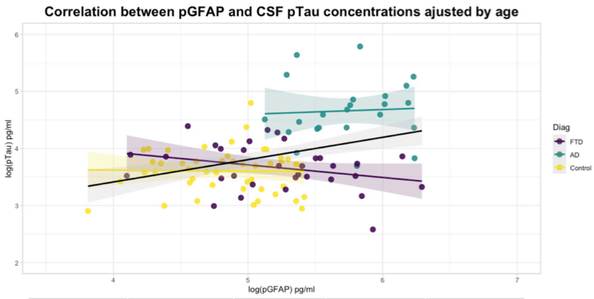

	All Group	FTD	AD	CN
Rho (p value)	0.33 (p=0.001)	-0.28 (p=0.12))	0.12 (p=0.59)	-0.001 (p=0.9)

Correlation of pGFAP with CSF Aβ42/Aβ40 ratio (A), Amyloid-PET centiloid (B), CSF total Tau (C) and CSF phosphorylated Tau (D) in the whole sample, FTD, AD and normal cognitively. All the correlation analysis are adjusted by age and sex. Abbreviations: pGFAP = plasma Glial fibrillary acidic protein; AB42/AB40 = Aβ42/Aβ40 ratio; tTau = total Tau; pTau = phosphorylated Tau; FTD = Frontotemporal dementia; AD = Alzheimer’s Disease; CN = Cognitively normal participants
